# Mining of disease-resistance genes in *Crocus sativus* based on transcriptome sequencing

**DOI:** 10.3389/fgene.2024.1349626

**Published:** 2024-02-02

**Authors:** Dongdong Ye, Siwei Zhang, Xiankui Gao, Xiujuan Li, Xin Jin, Min Shi, Guoyin Kai, Wei Zhou

**Affiliations:** ^1^ Zhejiang Provincial TCM Key Laboratory of Chinese Medicine Resource Innovation and Transformation, School of Pharmaceutical Sciences, Zhejiang Chinese Medical University, Hangzhou, China; ^2^ State Key Laboratory of Subtropical Silviculture, Zhejiang A&F University, Hangzhou, Zhejiang, China

**Keywords:** *Crocus sativus* L, transcriptome sequencing, *de novo* assembly, disease resistance gene, transcription factors

## Abstract

**Introduction:** *Crocus sativus* L. has an important medicinal and economic value in traditional perennial Chinese medicine. However, due to its unique growth characteristics, during cultivation it is highly susceptible to disease. The absence of effective resistance genes restricts us to breed new resistant varieties of *C. sativus*.

**Methods:** In present study, comprehensive transcriptome sequencing was introduced to explore the disease resistance of the candidate gene in healthy and corm rot-infected *C. sativus*.

**Results and discussion:** Totally, 43.72 Gb of clean data was obtained from the assembly to generate 65,337 unigenes. By comparing the gene expression levels, 7,575 differentially expressed genes (DEGs) were primarily screened. A majority of the DEGs were completely in charge of defense and metabolism, and 152 of them were annotated as pathogen recognition genes (PRGs) based on the PGRdb dataset. The expression of some transcription factors including NAC, MYB, and WRKY members, changed significantly based on the dataset of transcriptome sequencing. Therefore, this study provides us some valuable information for exploring candidate genes involved in the disease resistance in *C. sativus*.

## 1 Introduction

*Crocus sativus* L., also known as saffron, is the dried stigma of a perennial bulbous herb of the genus *Crocus* in Iris family (Nemati et al., 2018). As an auto-triploid sterile plant, lack of fertile seeds limits its reproduction of saffron. Therefore, it only relies on the corm for vegetative reproduction, which is beneficial to maintain its genetic characteristics, but limits its improvement in variety (Taheri-Dehkordi et al., 2020). Saffron can produce a large number of unique compounds including crocin, crocetin, carotenoids, flavonoid glycosides, volatile oil, phenolic acid and glycoside, and it is widely used in medicine, dyes, spices and food additives (Baba et al., 2015; Amini et al., 2017). Modern pharmacological experiments have shown that crocin and its derivatives show multiple pharmacological functions, including hyperlipidemia precautionary effect, antioxidant activity, immune regulation, and have been shown to have excellent effects on depression, anxiety, obsessive-compulsive disorder, insomnia, and other neuropsychiatric disorders (Lunt and Sondheimer, 1949; Escribano et al., 1999; Bathaie and Mousavi, 2010; Kamalipour and Akhondzadeh, 2011; Hoshyar and Mollaei, 2017; Rahaiee et al., 2017). Its outstanding pharmacological effects and excellent plant characteristics render saffron as one of the world’s most precious flower varieties, known as “plant gold”. However, saffron is susceptible to corm rot in much of its production area (Han et al., 2019).

Throughout their growth and development, plants are often plagued by foreign invaders, e.g., pathogenic microorganisms. In the long process of resistance evolution, plants gradually develop a multi-level set of coping and defense mechanisms (Wiesner-Hanks and Nelson, 2016). When suffering from pathogens, plants employ a two-layered innate immune responses to resist attacks. The first layer of the immune system is pattern-triggered immunity (PTI) through initiating by cell surface-localized pattern-recognition receptors (PRRs). Another is effect-triggered immunity (ETI) activated by intracellular receptors. PTI is triggered upon recognition of the pathogen-associated molecular patterns (PAMPs) via cell surface localized PRRs. PTI is thought to play a vital role in resisting pathogen invasion and maintaining the balance of microbial populations in plant leaves (Niehl et al., 2016; Yuan et al., 2021). To accelerate invasion, a lot of pathogens secrete virulence-associated molecules, e.g., effectors, into plant cells or the apoplast to interfere with host immunity. To combat the virulence of pathogens, plants often activate a second immune signaling known as ETI (Lahaye, 2002; Calle García et al., 2022). The activation mechanism of PTI and ETI immune responses is based on specific receptors encoded by the so-called pathogen recognition genes (PRGs). PRGs can be functionally grouped in seven distinct classes based on the presence of specific domains. The CNL class comprises a coiled-coil domain, a nucleotide binding site and a leucine-rich repeat; the TNL class comprises a Toll-interleukin receptor-like domain, a nucleotide binding site and a leucine-rich repeat. Moreover, the other classes contain the receptor-like kinase (RLK), the receptor-like protein (RLP), lysin motif receptor kinase (LYK), lysin motif protein (LYP) and lectin-receptor kinase (LECRK) (Liu et al., 2007; Zhang et al., 2021; Calle Garcia et al., 2022).

It has been validated that multiple signaling pathways are involved in stress-resistant immune mechanisms in plants (Ding et al., 2022). Plant mitogen-activated protein kinase (MAPK) modules play a key role in the induction of defense mechanisms by amplifying and transducing pathogen-derived signals perceived by membrane receptors (Pitzschke et al., 2009). In addition, calmodulin-like proteins (CML) can recognize calcium signals and regulate calcium ion levels to promote the production of reactive oxygen and enhance hypersensitive response (HR) (Köster et al., 2022). Salicylic acid (SA) and jasmonic acid (JA) are thought to be the main defense hormones. In special, SA is an important component of systemic-acquired resistance. Other hormones related to plant growth, such as auxin and abscisic acid (ABA), can also affect plant immune response (Berens et al., 2017).

Transcription factors (TFs) act as important regulatory systems in regulating the transcriptional expression of downstream genes by specifically binding to *cis*-elements in the promoters of downstream target genes, thus giving them the potential to regulate signaling cascades in the organisms. TFs have been validated to act as an indispensable role when plants sense a threat (Kesarwani et al., 2007; Goto et al., 2015). The *WRKY* gene family is one of the largest TF families in plants. Several members of the *WRKY* genes have been shown to bind with W-box, serving as a *cis*-acting element to modulate the transcription of many plant defensive genes. In rice, *WRKY45* is validated to participate in plant immune response, as a positive regulator (Goto et al., 2015). The TGACG motif-binding (TGA) class of bZIP TFs have also been identified in association with plant disease resistance. Other TF families including MYB, AP2/ERF, and bHLH, can form complex regulatory networks during plant stress (Kesarwani et al., 2007).

The development and application of high-throughput sequencing technologies has provided us a new perspective for studying complex biological processes. This strategy has been extensively introduced to identify the related functional genes participating in disease resistance in many medicinal plants, such as *Panax notoginsen*, *Panax ginseng*, and so on (Zhen et al., 2015; Ning et al., 2021). By transcriptomic analysis of resistant and susceptible *P. notoginseng* genotypes to *Fusarium oxysporum* infection, thirty PRGs were identified in association with root rot resistance in *P. notoginsen.* Moreover, SA and JA signal transduction genes together with *NAC* and *WRKY* family members involved in disease defense were identified (Ning et al., 2021). Based on comparative data of root transcriptomes from different varieties of *P. ginseng*, 28 PRGs contributing to better disease resistance and cytochrome p450s together with glycosyltransferases related to higher ginsenoside content in the wild, were identified (Zhen et al., 2015). However, little data has been presented on the disease-resistant genes in saffron. In present study, RNA sequencing (RNA-Seq) technology was employed to conduct a comprehensive transcriptome analysis of healthy and corm rot-infected saffron, to predict the candidate genes involved in disease resistance, providing valuable data for studying disease resistance in saffron.

## 2 Materials and methods

### 2.1 Plant materials

Saffron corms were collected from Jiande City, Zhejiang province, China. According to surface rot, the collected saffron corms were divided into healthy and infected groups, labeled as *C. sativus* healthy groups (CsHG, no rot phenotype) and *C. sativus* infected groups (CsIG, surface rot rate >25%), respectively (Supplementary Figure S1). All collected corms were frozen in liquid nitrogen immediately and kept at −80°C until use.

### 2.2 RNA preparation and sequencing

Six total RNA from healthy and rot-infected saffron corms with three biological replicates were isolated using the *TRI*zol reagent (Invitrogen), respectively. The integrity of RNA was firstly determined using EtBr-stained 1% agarose gel. RNA purity and concentration were further detected by using a Nanodrop spectrophotometer (Thermo) (Zhou et al., 2017). By utilizing magnetic beads with Oligo (dT) to pair the A-T bases with ployA, the mRNA was further extracted from total RNA for conducting transcriptome sequencing.

Based on the Illumina Novaseq 6000 platform, six-base random hexamers were added by using the reverse transcriptase to inversely synthesize cDNA strand using the six mRNA extracts as template, respectively. Following this, six cDNA libraries were constructed. Finally, Illumina sequencing platform was introduced to perform transcriptome sequencing of the six samples. The Illumina transcriptome sequencing data (accession number PRJNA1005600) was deposited in the Genome Sequence Archive at the Beijing Institute of Genomics Data Center (https://bigd.big.ac.cn/), Chinese Academy of Sciences.

### 2.3 *De novo* assembly and functional annotation

In the absence of a publicly available reference genome of *C. sativus*, high-quality RNA-seq data was obtained, by removing the adaptor sequences and repeated, and low-quality reads, to obtain clean reads. We used the Trinity (https://github.com/trinityrnaseq/trinityrnaseq/wiki) to get a high quality of clean data, assembled from scratch, thus producing transcripts and unigenes of *C. sativus* (Grabherr et al., 2011). All unigenes have been assembled based on the NCBI non-redundant protein database (NR, ftp://ftp.ncbi.nlm.nih.gov/blast/db/), the Pfam (http://pfam.xfam.org/), the Swiss-Prot (http://web.expasy.org/docs/swiss-prot_guideline.html), the Gene Ontology database (GO, http://www.geneontology.org), the Clusters of Orthologous Groups (COG, http://www.ncbi.nlm.nih.gov/COG/), and the Kyoto Encyclopedia of Genes and Genomes (KEGG, http://www.genome.jp/kegg), using BLASTP with an E-value cutoff of 1e^−5^. The aligned hits with at least 50% coverage of seed protein sequences and >50% protein sequence identity were selected as homologs to obtain annotation and classification information in each database (Altschul et al., 1997). Plant Transcription Factor Database had been introduced to identify the candidate unigenes of TFs (Tian et al., 2020).

### 2.4 Gene expression analysis

We use RSEM to conduct quantitative expression analysis of all unigenes, and Transcripts Per Million reads (TPM) was introduced to calculate the transcripts of each unigene (Li and Dewey, 2011). The disease resistance candidate unigenes exhibiting variant expression levels were selected with the DESeq2 software between healthy and infected groups with *p*-value <0.05 and |log_2_ Fold Change (FC)| ≥ 2 as a cutoff. The fact that lower *p*-value and larger log_2_FC absolute value was more likely to obtain disease resistance gene (Zhen et al., 2015). In order to detect the probability of errors in the overall inference results, FDR correction with Benjamini/Hochberg (BH) was introduced to conduct multiple inspections and corrections for the *p*-value obtained by statistical inspection (Nawaz et al., 2019). GO functional enrichment and KEGG pathway analysis were employed by Goatools and KOBAS, respectively (Xie et al., 2011).

### 2.5 Mining of pathogen recognition genes

The Plant Resistance Genes Database (PRGdb, http://prgdb.org) is a community-based database to study genes involved in pathogen recognition genes (PRGs) (Sanseverino et al., 2010). We employed PRGdb to explore the PRGs. The Disease Resistance Analysis and Gene Orthology (DRAGO 3) is an online tool for automatic annotation and prediction of PRGs behind PRGdb. We downloaded the protein sequences of all of the curated resistance genes from this website and used BLASTX to search our assembled transcriptome for the protein sequences (cutoff E-value: 1×10^−20^). HMMER v3 package was used to identify the LRR, Kinase, NBS, and Toll-interleukin receptor-like (TIR) domains in the candidate pathogen resistance genes from 60 HMM modules (Johnson et al., 2010), and COILS 2.2 and TMHMM 2.0c programs were employed to identify CC and TM domains in the candidate pathogen resistance genes (Osuna-Cruz et al., 2018). HMMs were further filtered with hmmsearch (default parameters; hmmer tool; http://hmmer.org/) against the initial FASTA files to test whether they were indeed useful for resistance domain prediction.

### 2.6 Quantitative real-time PCR detection

To verify the RNA-Seq results for gene expression exhibited by the TPM values, candidate genes were picked out for quantitative real-time PCR (qRT-PCR) analysis. Total RNA was isolated using the *TRI*zol reagent, and the first cDNA strand was synthesized by reverse transcriptase using the PrimeScript™RT Reagent Kit (Takara, Japan). Real-time PCR was performed using SYBR Pre-mix Ex Taq™ II kit (Promega, Beijing). The reagent volume was finally fixed to 10 mL, including 1 μL cDNA template, forward primer 0.2 μL, reverse primer 0.2 μL, SYBR^®^ Premix Ex *Taq*™ II (Tli RNasEH Plus) (2×) 5 μL and ddH_2_O 3.6 μL. The relative expression levels of the candidate genes were calculated in relation to the reference gene *CsActin* by the 2^−ΔΔCT^ method (Livak and Schmittgen, 2001). The primer sequences used for qRT-PCR in present study are listed in Supplementary Table S1. Each generated data represents the average value of three biological replicates, and the data are expressed as the mean ± SD. SPSS 16.0 software was used to analyze statistical significance by single sample *t*-test and one-way analysis of variance, and *p*-values <0.05 were considered statistically significant.

## 3 Results

### 3.1 Transcriptome sequencing and *de novo* assembly

**FIGURE 1:**
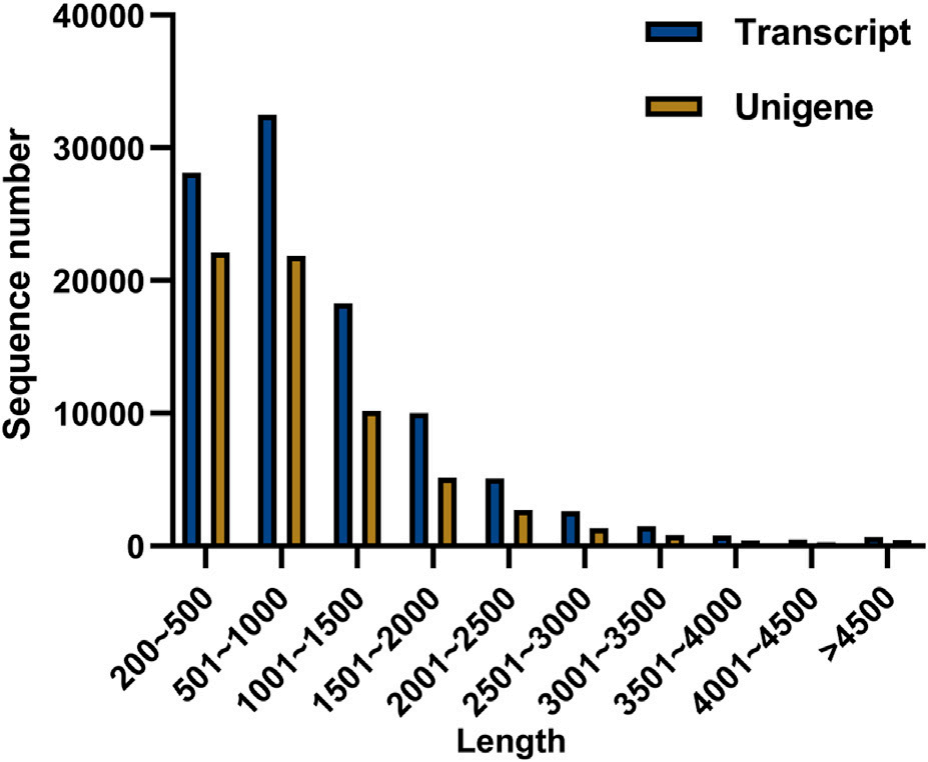
Distribution of different lengths of transcripts and unigenes.

To explore the candidate genes associated with pathogen resistance, three infected saffron corms and three healthy ones were collected for transcriptome sequencing. cDNA libraries of the six samples were constructed and sequenced using Illumina double-terminal sequencing technology. Thus, 53,092,375 mean raw reads were generated from the infected saffron corms and 45,552,810 mean raw reads were collected from the healthy samples. After removing the adapter sequences, low quality reads, and short reads, approximately 52,799,054 and 45,330,599 mean clean reads were obtained from the infected and healthy corms, respectively, generating a total of 43.72 Gb of clean data for the six samples (Table 1). High-quality clean reads were assembled using the Trinity process, resulting in the identification of 100,011 transcripts and 65,337 unigenes. The length distribution of 28% of the transcripts and 34% of unigenes was greater than 1 kb (Table 2; Figure 1). The average length of a unigene was 955.1 bp, and the N50 length was 1,291 bp (Table 2).

**TABLE 1:** Summary of a *C. sativus* transcriptome.

Sample	Raw reads	Raw bases	Clean reads	Clean bases	Error rate (%)	Q20 (%)	Q30 (%)	GC content (%)
CsHG1	42202774	6330416100	42036298	6274622188	0.0264	97.52	92.84	46.42
CsHG2	50806370	7620955500	50547358	7525702935	0.0269	97.31	92.38	47.84
CsHG3	43649286	6547392900	43408142	6463228851	0.0269	97.29	92.37	47.92
CsIG2	61955334	9293300100	61619416	9051293836	0.0258	97.73	93.46	46.73
CsIG3	44154018	6623102700	43911088	6512439403	0.0260	97.60	93.21	47.97
CsIG4	53167774	7975166100	52866660	7891077680	0.0258	97.69	93.41	49.38

**TABLE 2:** Summary of results of the sequence assembly.

Type	Unigenes	Transcripts
Total number	65337	100011
Total base	62403351	105240656
Largest length (bp)	15573	15573
Smallest length (bp)	201	201
Average length (bp)	955.1	1052.29
N50 length (bp)	1291	1420
Fragment mapped percent (%)	59.272	76.083
GC percent (%)	45.51	45.27
BUSCO score	76.5%	81.0%

### 3.2 Functional annotation and classification of unigenes

**FIGURE 2:**
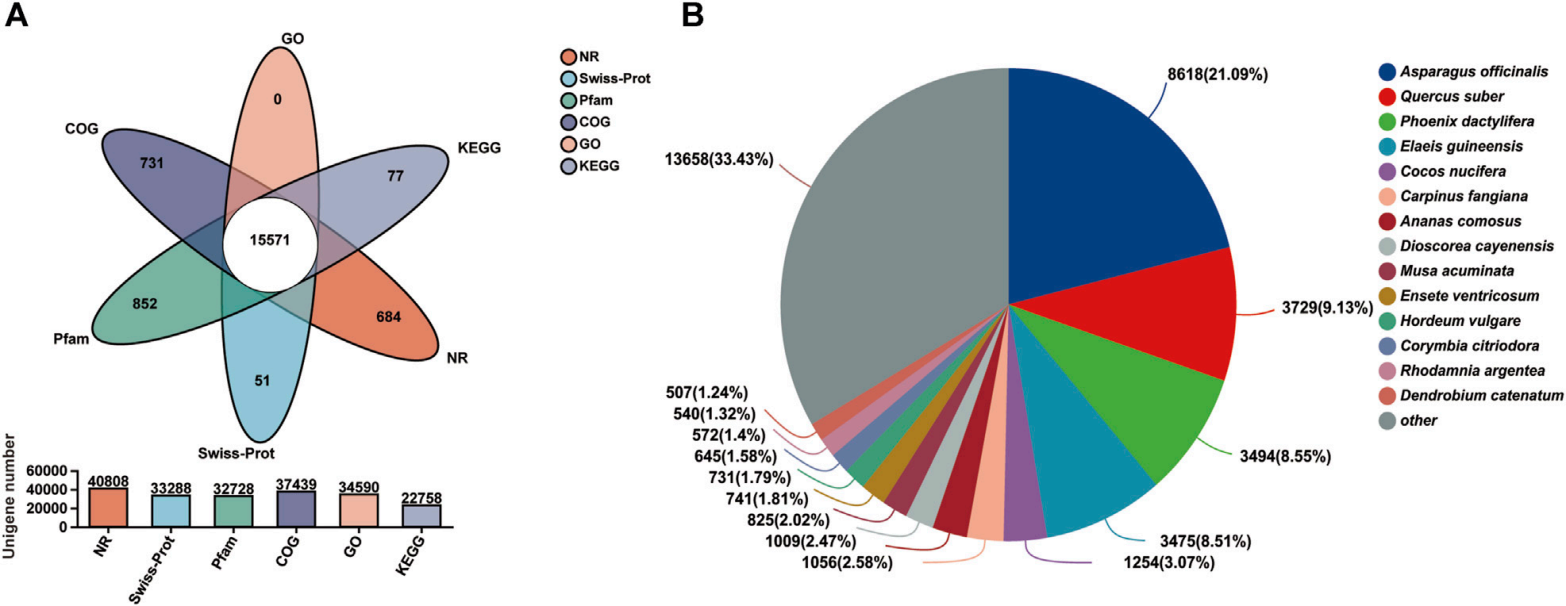
Transcriptome functional annotation. **(A)** Venn diagram of the functional annotation upon the six public protein databases (NR, Pfam, Swiss-Prot, GO, COG, and KEGG). **(B)** Distribution of the matching homologous species based on NR annotation.

A total of 43,834 unigenes occupying 67.11% of the total assembled unigenes were successfully annotated by a similarity search within the NR, GO, COG, Pfam, Swiss-Prot, and the KEGG databases, respectively (Table 3; Figure 2A) (Grabherr et al., 2011). Among them, 15,571 unigenes matched the known sequences in more than one database; whereas, 684, 731, 51, 852, and 77 uingenes were annotated as unique ensembles in the five public databases including NR, COG, Pfam, Swiss-Prot and KEGG, respectively (Figure 2A). In the NR database, 8,618 unigenes were closest to the gene sequences of *Asparagus officinalis*, ranging from 80% to 100% in sequence similarity (Figure 2B; Supplementary Figure S2).

**TABLE 3:** Functional annotation of unigenes in the six public databases.

	Unigene number (percent)	Transcript number (percent)
GO	34590(0.5302)	59049(0.5924)
KEGG	22758(0.3488)	38204(0.3833)
COG	37439(0.5738)	64435(0.6464)
NR	40808(0.6255)	69163(0.6938)
Swiss-Prot	33288(0.5102)	56592(0.5677)
Pfam	32728(0.5016)	55347(0.5552)
Total annotation	43788(0.6711)	72363(0.7259)
Total number	65244(1)	99682(1)

Based on gene ontology annotations, all unigenes can be classified into three categories: biological processes, cellular components, and molecular functions (Supplementary Figure S3; Supplementary Table S2). Biological processes were then identified through the Kyoto encyclopedia of genes and genome (KEGG) database and six KEGG classes, and 20 pathways were identified (Supplementary Figure S4; Supplementary Table S3). According to the sequence homology, 37,475 unigenes were grouped into four COG classes. Of these, 214 unigenes were attributed to disease defense mechanisms and were predicted to be associated with the disease defense of saffron (Supplementary Figure S5; Supplementary Table S4).

### 3.3 Gene expression and enrichment analysis

**FIGURE 3:**
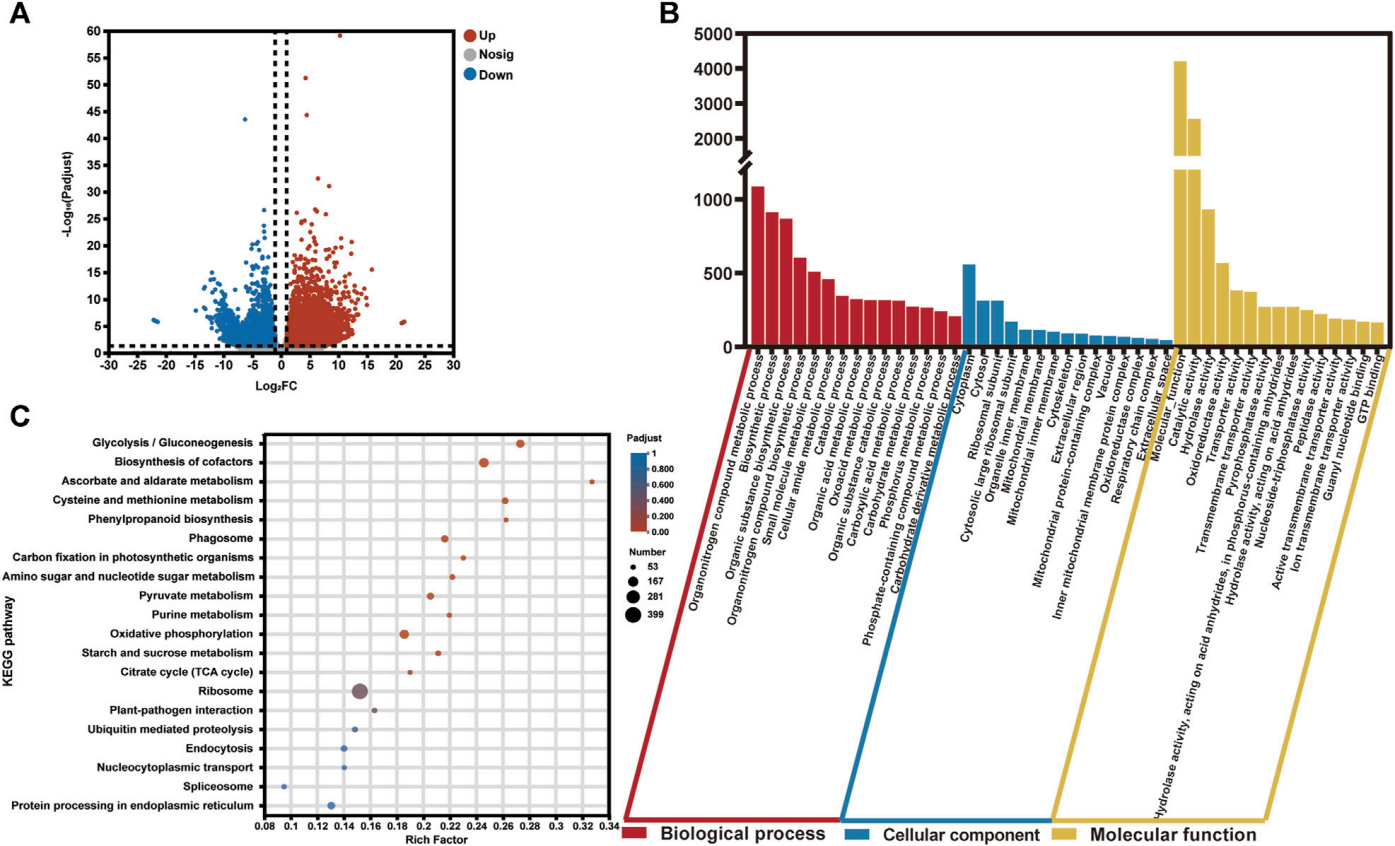
Statistical and enrichment analysis of differentially expressed genes in the healthy and susceptible groups of *C. sativus.* **(A)** *P*-adjust <0.01 and the absolute value of |log_2_FC| ≥2 were used as a cutoff to select the DEGs in the transcriptome data. **(B)** GO enrichment analysis of DEGs. **(C)** KEGG pathway classification and functional enrichment of DEGs.

To investigate the differentially expressed genes (DEGs) in the two groups (healthy and infected) of saffron corms, we calculated the normalized expression values of the Transcripts Per Million (TPM) reads of each unigene. In total, 7,575 DEGs showed significantly variant expression patterns between the healthy and rot-infected corms, of which, 4,416 unigenes were upregulated and 3,159 unigenes were downregulated in the rot-infected saffron corms (Figure 3A; Supplementary Table S5). A cluster analysis of the gene expression profiles of all DEGs revealed that the infected group (CsIG) of saffron corms showed significantly variant gene expression during disease invasion relative to the healthy group (CsHG) (Supplementary Figure S6).

The main functional categories of DEGs were classified using gene ontology (GO) enrichment analysis. The functions were divided into three types, including biological processes, cellular components, and molecular functions. We observed that for all the DEGs, the top three classes that contributed to “biological processes” were, the organonitrogen compound metabolic process (1,087), the biosynthetic process (911), and the organic substance biosynthetic process (868). In the cellular component function category, the top three classes consisted of the cytoplasm (559), cytosol (312), and ribosomal subunit (312). Although, in terms of the molecular function category, the molecular function (4,205) enriched most of the unigenes. This was followed by the catalytic (2,555) and hydrolase activities (933) (Figure 3B; Supplementary Table S6).

Subsequently, a total of 3,646 DEGs were retrieved from the Kyoto Encyclopedia of Genes and Genomes (KEGG) database; 127 KEGG pathways were enriched, which predominantly included “Genetic Information Processing,” “Metabolism,” “Environmental Information Processing,” “Cellular Processes,” and “Organismal Systems.” Among them, the top five pathways with the highest enrichment number of DEGs were ribosome (399), biosynthesis of cofactors (157), oxidative phosphorylation (147), glycolysis/gluconeogenesis (118), and protein processing in the endoplasmic reticulum (106) (Figure 3C; Supplementary Table S7). At the same time, we found that among the 127 KEGG pathways, there were many enrichment pathways related to plant disease, including oxidative phosphorylation (147), plant-pathogen interaction (62), phenylpropanoid biosynthesis (53), plant hormone signal transduction (45), glutathione metabolism (44), and the MAPK signaling pathway (43). The GO and KEGG annotations of the DEGs provide us useful information to identify specific pathways and related genes in association with disease resistance to corm rot in saffron.

### 3.4 DEGs involved in plant hormone signal transduction and plant-pathogen interaction

**FIGURE 4:**
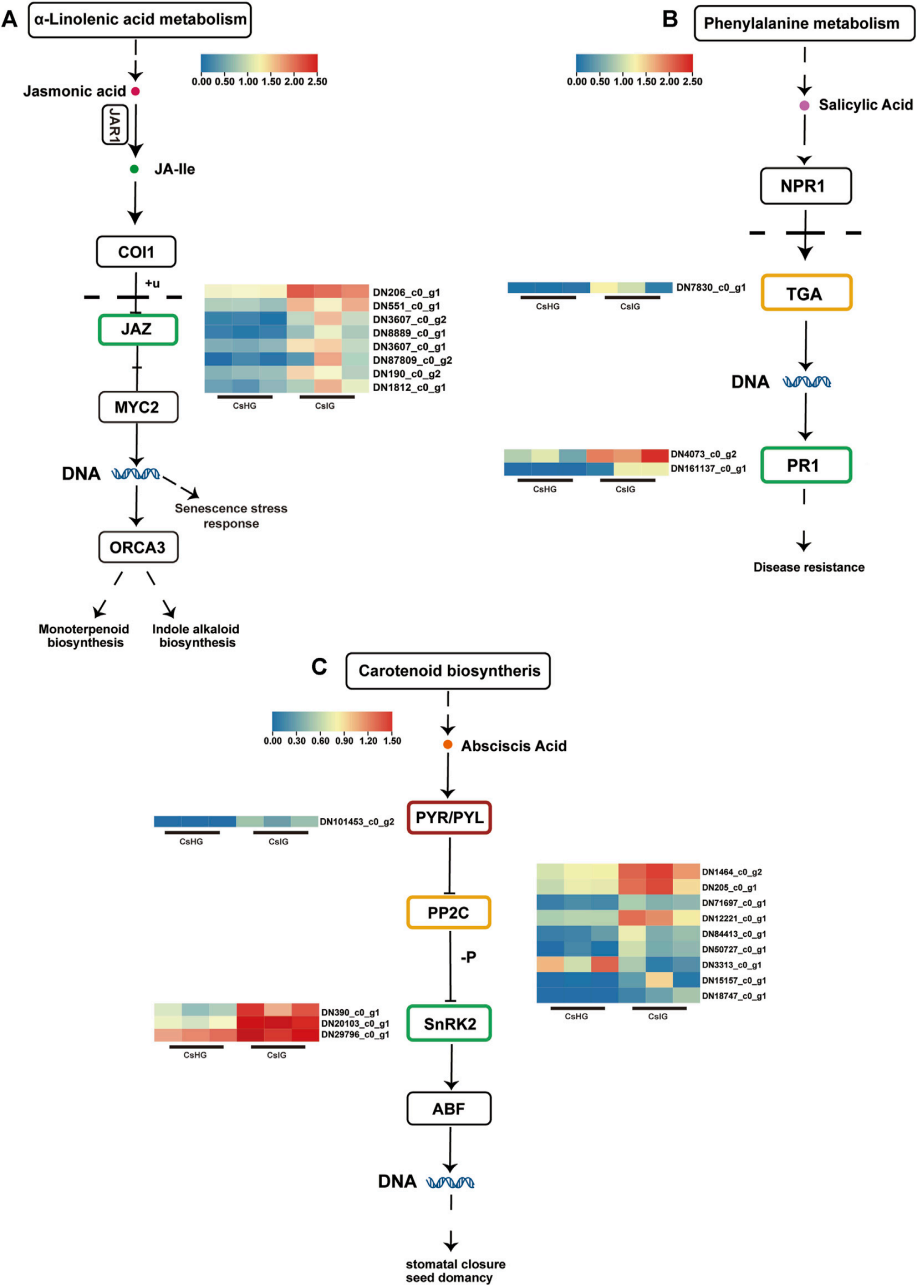
DEGs associated with plant hormone signal transduction pathways. **(A)** Jasmonic acid signal transduction pathway. **(B)** Salicylic acid signal transduction pathway. **(C)** Abscisic acid signal transduction pathway. Expression values are labeled as log_10_ TPM values.

When plants are subjected to various biotic or abiotic stresses, local cells produce corresponding chemical signals to induce the expression of hormone-related genes thus regulating immune responses (Robert-Seilaniantz et al., 2011). In order to determine whether saffron activates relevant hormone-responsive genes to participate in defense against threats during pathogen stress, we analyzed the expression of several hormone signaling related genes. It was revealed that 24 DEGs were involved in the plant hormone pathways (Figure 4; Supplementary Table S8). Jasmonic acid (JA) played an important role in regulating plant growth and development. We found that eight jasmonate zim-domains (JAZ) genes involved in JA signal transduction were upregulated in the disease group (Figure 4A). As the core receptor of salicylic acid (SA), the non-expressor of pathogenesis-related (PR) genes 1 could activate the expression of TGACG motif-binding transcription factor (TGA) when the SA content increased; thereby it activated the expression of the *PR1* gene, to enhance disease resistance (Chen et al., 2021). When faced with disease invasion, the expression of the *TGA* transcription factor (DN7830_c0_g1) was significantly increased, while the expression of the downstream *PR1* gene (DN4073_c0_g2, DN161137_c0_g1) was significantly upregulated, compared to the healthy group (Figure 4B). Meanwhile, the expression levels of ten genes, identified as abscisic acid (ABA)-associated genes including nine protein phosphatase 2C (PP2C) genes, three *SnRK2* genes, and one *PYL4* gene, were increased to varying degrees after suffering with the disease (Figure 4C). These results suggest that JA, SA, and ABA could be involved in the disease resistance of saffron.

**FIGURE 5:**
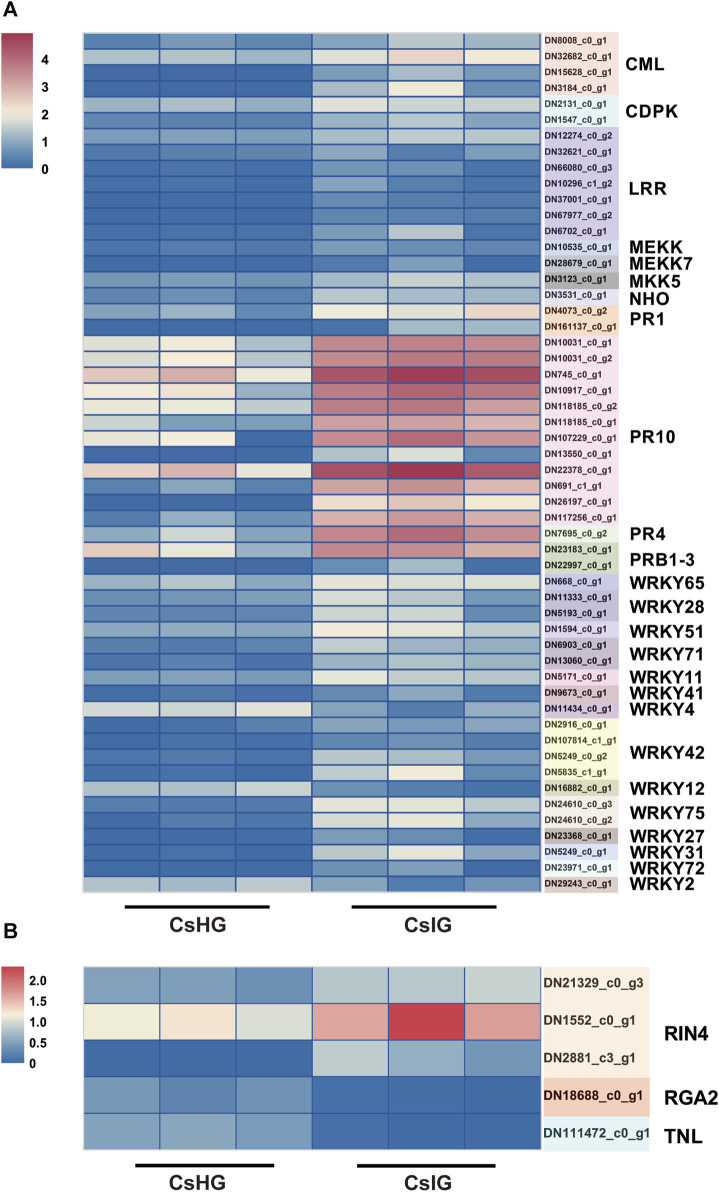
Analysis of DEGs associated with the plant-pathogen interaction pathways. **(A)** DEGs involved in the PTI immunity pathway. **(B)** DEGs involved in the ETI immunity pathway. Expression values are labeled as log_10_ TPM values.

PTI, as a plant immune response, could be rapidly activate when it sensed a pathogen. Co-expressed with ETI, the immune response could be promoted and programmed cell death (PCD) is triggered, for which it limited the spread of pathogens, by triggering a hypersensitive response (HR) in plants (Chen et al., 2021). Therefore, we investigated the expression of these immune pathway-related genes, and 55 DEGs involved in the PTI pathway, including four that were calmodulin-like (CML), two calcium-dependent protein kinases (CDPK), two mitogen extracellular signal-regulated kinase kinase (MEKK), one glycerol kinase (NHO), two PR protein 1, and seven LRRs were identified (Figure 5A) (Bigeard et al., 2015). In addition, five DEGs participating in the ETI pathway were picked out, including one resistance gene analog 2 (RGA2), one TNL, and three RPM1-interacting protein 4 (RIN4) genes (Supplementary Table S9) (Yuan et al., 2021). The expression level of two CDPK genes (DN2131_c0_g1, DN1547_c0_g1) in association with the cell-wall strengthening and allergic reaction induced by the immune process (Lincoln et al., 2018), was significantly increased in corm rot-infected saffron. The RIN4 was thought to regulate the plant immunity and was validated to be a target of many disease-resistant proteins (Prokchorchik et al., 2020). In present study, we revealed that three RIN4 genes (DN21329_c0_g3, DN1552_c0_g1, DN2881_c3_g1) were simultaneously upregulated in the infected corm (Figure 5B).

### 3.5 Identification of pathogen recognition genes and detection of the gene expression profiles

**FIGURE 6:**
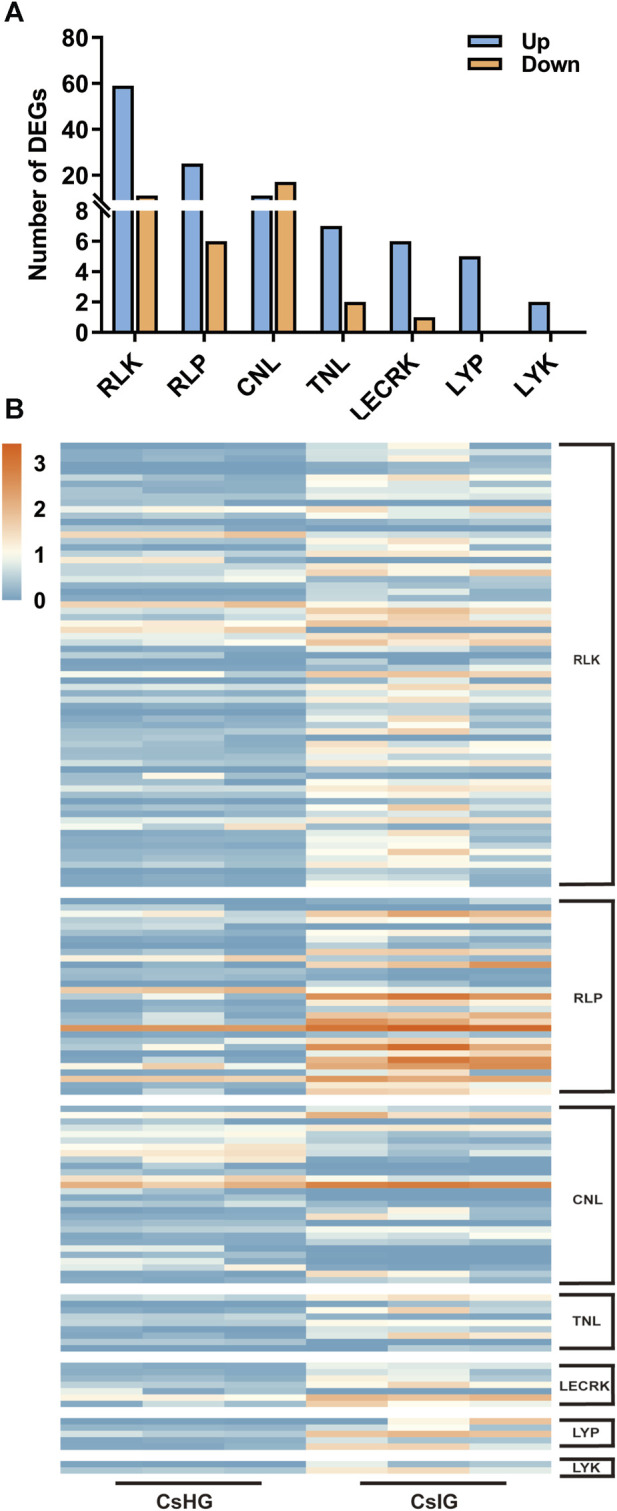
Classification and expression analysis of pathogen recognition genes (PRGs). **(A)** Classification of PRGs. **(B)** Expression profiles of PRGs.

**FIGURE 7:**
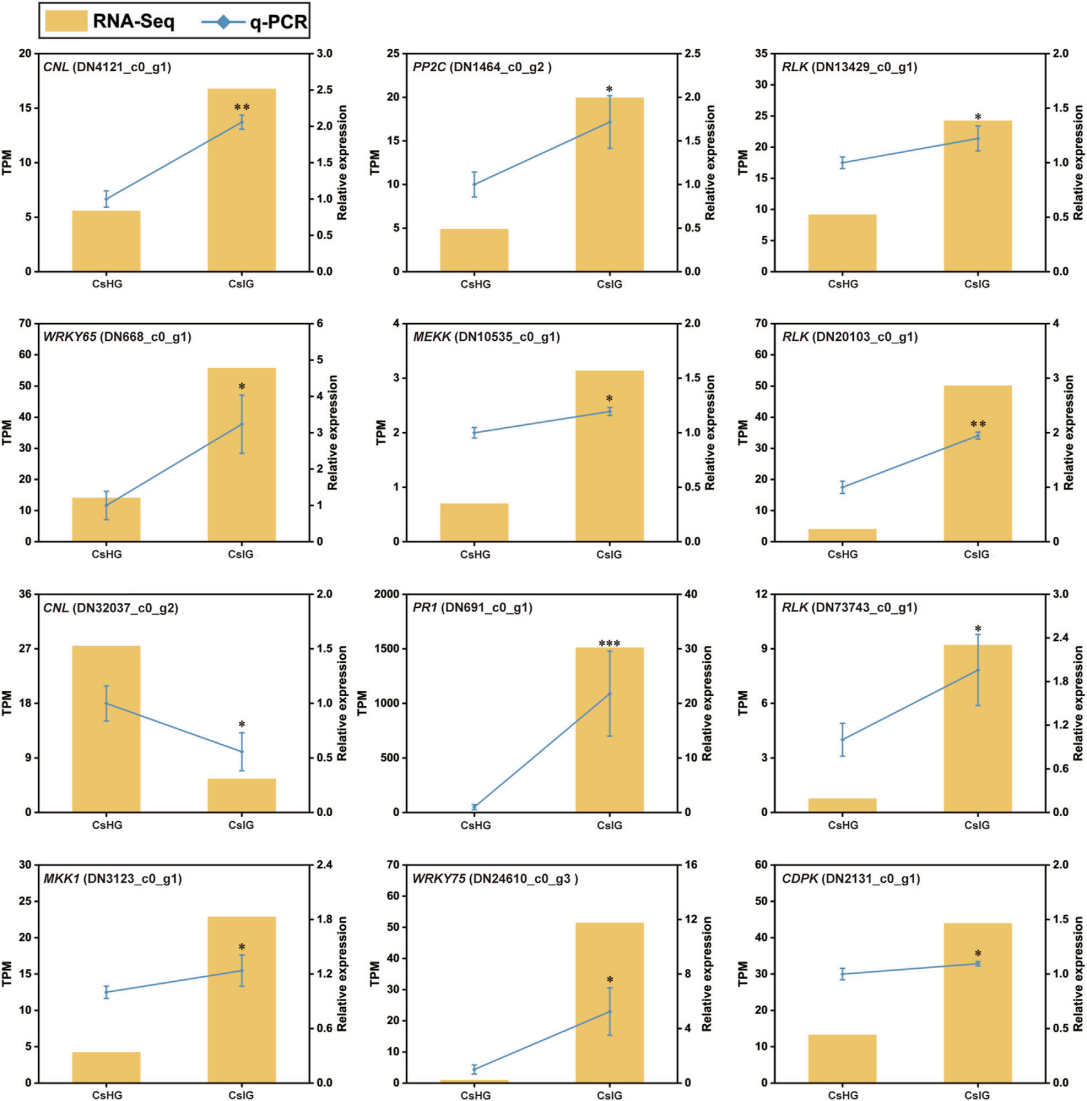
qRT-PCR analysis of differentially expressed pathogen recognition genes associated with disease resistance in *C. sativus*. Error bars represent ± SD of triplicates for qRT-PCR validation. Asterisk indicates significant differences at one significant level of *t*-test (*p < 0.05; **p < 0.01)

The pathogen recognition genes (PRGs) can sense and recognize plant pathogen invasion and activate immune through the PTI and ETI immune system to resist pathogen invasion (Yuan et al., 2021). PRGs can be functionally divided into seven distinct subgroups based on the presence of specific domains including CNL, TNL, RLK, RLP, LYK, LYP and LECRK subgroups (Calle Garcia et al., 2022). To mine the PRGs in saffron, we collected the DEGs between the healthy and corm rot-infected groups, and mapped them to the PRGdb database, which is a web-accessible open source database (http://www.prgdb.org) and is valuable for us to annotate the PRGs of plant. In total, 152 DEGs were annotated as seven different PRGs classes (Supplementary Table S10). Compared to healthy saffron, 115 PRGs were upregulated and 37 of them were downregulated after infection by the disease (Figures 6A, B). To confirm the reliability of the transcriptome data, qRT-PCR detection was introduced to verify the expression profiles of DEGs obtained from the transcriptome dataset. In the present study, 12 disease-resistance genes were randomly selected for qRT-PCR validation, including *PR1*, *CDPK*, *PP2C*, *MEKK*, *MKK1*, *WRKY65*, *WRKY75*, and five PRGs. The results showed that the expression profiles of these genes validated by qRT-PCR were in accordance with the RNA-Seq results, implying that the database collected by RNA-Seq in this study was reliable (Figure 7).

### 3.6 Mining of the candidate transcription factors involved in disease resistance

**FIGURE 8:**
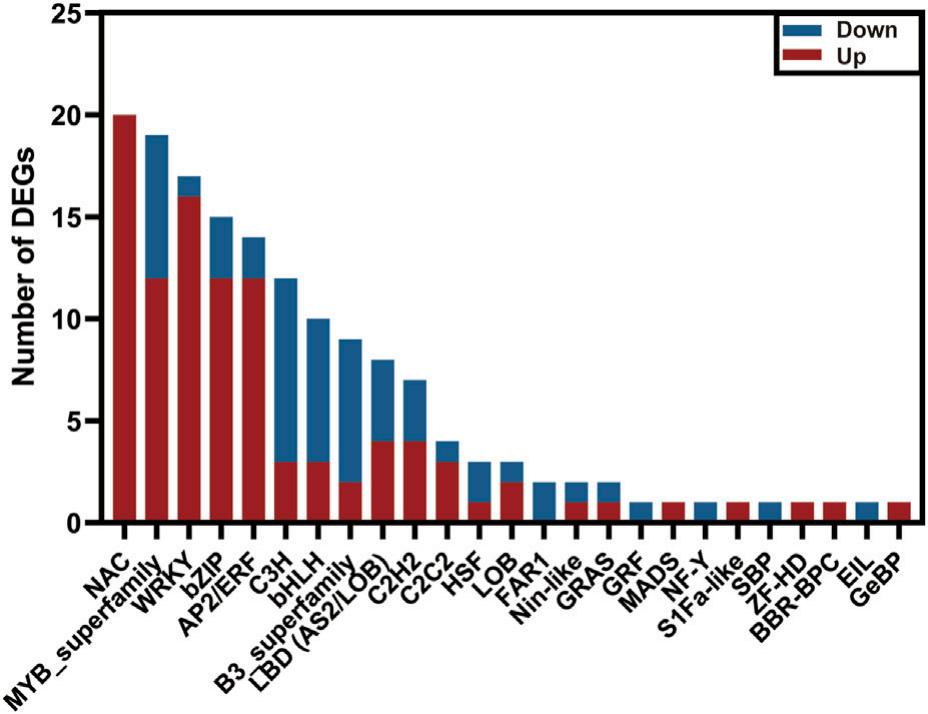
The number of upregulated and downregulated transcription factors in the transcriptome data of *C. sativus*.

Transcription factors (TFs) act as a crucial role in regulating plant tolerance to pathogen infection (Bedoya et al., 2012; Yao et al., 2019). By homologous comparisons with Plant TFDB, approximately 1,045 unigenes out of the above 7,575 DEGs were annotated as TFs, representing 34 families. Of these, the MYB family contained the largest number of TFs, up to 143 members, followed by AP2/ERF (102), bZIP (77), and bHLH families (69) (Supplementary Figure S7). To explore the candidate TFs resisting extrinsic disease stress in *C. sativus*, we investigated the expression profiles of the 1,045 TFs between healthy and rot-infected corm based on the transcriptome dataset. In total, 156 TFs exhibiting significant differential expression patterns were mined, and they were classified into 25TF families. Among them, 101TFs were upregulated, whereas the other 55TFs were downregulated. The NAC family (20) had the largest number of members, followed by the MYB (19), WRKY (17), bZIP (15), AP2/ERF (14), C3H (12) and bHLH families (10) (Figure 8). Previous studies have shown that WRKY transcription factors played a vital regulatory function in immune responses (Kim et al., 2008; Chen et al., 2013; Xiao et al., 2023). In this study, a total of 17 WRKY TFs were validated to be upregulated and three WRKY TFs were downregulated in response to the disease invasion (Figure 5A).

## 4 Discussion

### 4.1 Hormonal regulation involved in disease resistance

Saffron is a traditional Chinese medicinal herb. However, saffron has been plagued by disease due to its asexual trait. One of the main causes that limits saffron development is corm rot (Han et al., 2019). Exploring the resistance genes in saffron is valuable for breeding new varieties. High-throughput sequencing technology provides us a good avenue to mine the candidate genes with regard to disease resistance in saffron (Zhen et al., 2015; Ning et al., 2021). In this study, we chose healthy and infected saffron corms for transcriptome sequencing to explore the candidate genes involved in disease resistance. In total, 7,575 differentially expressed genes were firstly screened, and the number of DEGs exhibiting an upregulation pattern (4,416) was more than the downregulated genes (3,159) (Supplementary Table S5). By KEGG pathway enrichment analysis, we revealed that DEGs in association with disease resistance were mainly congregated in plant-pathogen interaction (62), phenylpropanoid biosynthesis (53), plant hormone signal transduction (45), MAPK signaling pathway (43) and flavonoid biosynthesis (42) (Figure 3; Supplementary Table S7). Among them, phenylpropanoid and flavonoid was thought to play a vital role as precursors in the production of stress products for disease resistance when saffron corms suffered from disease invasion (Ramaroson et al., 2022; Xiao et al., 2023).

Jasmonic acid (JA), salicylic acid (SA), and abscisic acid (ABA), as three key signals to activate the plant immune response, play a vital role in emergency defense (Anderson et al., 2004; Yang et al., 2020). Therefore, in this study, 24 DEGs participating in the biosynthesis and signaling transduction pathways of JA, SA, and ABA in saffron, were paid more attention (Figure 4; Supplementary Table S8). JA is an important signal molecule for plant growth and coping with environmental stress. Jasmonate zim-domain (JAZ) is a family of transcriptional regulatory proteins that have been implicated in plant growth and defense responses (Oblessuc et al., 2020). With exposure to external stress, JA accumulates to induce the degradation of JAZ protein by the 26S proteasome, releasing JA-responsive transcription factors such as MYC2 to activate defense (Yang et al., 2020). In this study, we reveal eight JAZ unigenes are upregulated (Supplementary Table S8). SA signal induced by the pathogen is perceived by the receptor nonexpressor of pathogenesis-related genes 1 (NPR1), which is recruited by TGA transcription factors to induce the expression of PR genes. The expression of *PR1* gene has been used extensively as a marker for disease resistance in plants (Lincoln et al., 2018; Han et al., 2022). Moreover, SA has also been validated to directly promote the expression of *PR1* gene to resist pathogen invasion (Hu et al., 2022). TGA as an important regulatory factor in plant immunity, belongs to the basic leucine zipper transcription factor superfamily (Shimizu et al., 2022). We noted that the expression of a TGA transcription factor (DN7830_c0_g1) in SA signaling pathway predicting to be a target for PR protein, was significantly upregulated in corm rot-infected group (Supplementary Table S8) (Cox, 2022). ABA can induce stress response including stomatal closure, by binding to its receptor protein PYR/PYL and releasing protein phosphatase 2C (PP2C), thus inhibiting the activity of SNF1-related kinase 2 (SnRK2). Obviously, stomatal closure plays an important defense role in pathogen invasion (Lin et al., 2021). When saffron suffers from pathogen invasion, we find that one PYL4, nine PP2C, and three SnRK2 genes are almost all upregulated (Supplementary Table S8). Therefore, it is exciting to explore the mechanism of PYL4 and PP2C in response to pathogen invasion. Based on the above results, it is a reminder that saffron may defend disease invasion in a variety of integrated ways by activating various hormone-signaling genes.

### 4.2 Immune responses involved in disease resistance

The first major immune response, named PTI immune, is triggered when the pathogen breaks through the physical defense of the plant’s surface. Pattern recognition receptors (PPRs) on the plant cell surface recognize pathogen signals, leading to the first layer of inducible defenses (Yuan et al., 2021). PPRs in plants can be grouped into four types including RLK, RLP, LYP, and LYK. RLP only contain leucine-rich repeat region (LRR) and transmembrane domain (TM), whereas RLK in addition contain kinase domain (KIN) (Calle Garcia et al., 2022). At present, 70 DEGs have been annotated as RLK genes. The gene expression of 59 of them is elevated and of 11, is decreased. A total of 31 DEGs were annotated for RLP, of which 25 of them were upregulated (Figure 6; Supplementary Table S10). It’s worth noting that five of the RLPs (DN1802_c0_g1, DN26222_c0_g1, DN281_c0_g1, DN33904_c0_g1 and DN5708_c0_g1) were intensively induced in infected group (CsIG) compared to the healthy group (CsHG) with the increased value more than 50 folds, suggesting that they may play distinct contribution to corm rot disease resistance (Supplementary Table S10). As it is reported that many PRRs containing lysin motifs or lectin-like motifs, participate in defensing fungal pathogens (Calle Garcia et al., 2022). In rice, two lysin motif-containing proteins, OsLYP4 and OsLYP6, were validated to be the dual functional PRRs sensing bacterial peptidoglycan and fungal chitin to resist pathogen invasion (Liu et al., 2013). As a lysin motif receptor kinase (LYK), LYK5, is thought to be a major chitin receptor in *Arabidopsis* (Cao et al., 2014). In our study, the expression of 7 out of 14 genes identified as LYP and LYK was upregulated (Supplementary Table S10). This suggests that these genes may play an important role in the defense of fungal diseases in saffron. ETI immune is initiated following direct or indirect recognition of pathogen effectors by nucleotide-binding domain leucine-rich repeat containing receptors (Yuan et al., 2021). These receptors are intracellular and lead to a more robust immunity, which can be further divided into two categories: TNL and CNL (Calle Garcia et al., 2022), which have been validated to play a key role in plant disease resistance, such as *Arabidopsis* and maize (Tan et al., 2007; Xu et al., 2018). In our study, 33 of differentially expressed PRGs containing the domain of CNL and TNL were identified by comparison with seven PRGs involved in resisting disease in *Arabidopsis thaliana* (Tan et al., 2007), and one of the DEGs (DN19011_c0_g2) was highly homologous to AtCNL (AT4G26090). These results provide a more reliable basis for exploring candidate disease resistance genes (Supplementary Figure S8; Supplementary Table S12). The PRGs originate in the ancient immune system, and the evolution of PRGs in plants has a great genetic diversity. They play an irreplaceable role in pathogen resistance. To date, only some basic features of the PRGs have been revealed (Sanseverino et al., 2010). Of note, 152 PRGs have been screened from the transcriptome database, of which 115 have been implicated in PTI, and 37 in ETI (Figure 6; Supplementary Table S10). Therefore, we deduce that saffron may synchronously employ PTI and ETI immune reactions to resist disease invasion.

### 4.3 Transcription factors involved in disease resistance

In plants, Transcription factors (TFs) serve as positive or negative regulators, as they are involved in a variety of biotic and abiotic stresses, thus playing an important role in plant secondary metabolite pathways (Bedoya et al., 2012; An et al., 2020; Wang et al., 2021). In our study, the top five types of TFs exhibiting a significant variation of the gene expression level are NAC, MYB, WRKY, bZIP, and AP2/ERF, respectively (Supplementary Table S11). This is similar to the reported conclusions of Alves et al. (2014), suggesting that the 5 TF families may provide more contribution than other TF families, to adapt the plant defense response. NAC TFs act as transcriptional activations or repressions with a variable transcriptional regulatory region in the C-terminal region (Olsen et al., 2005). In *A. thaliana*, *NAC016* has been found to be involved in the drought stress response (Sakuraba et al., 2015). As a positive regulating factor in resisting rice blast and bacterial blight, overexpression of *NAC066* can enhance the disease resistance in rice (Liu et al., 2018). In our study, almost all the NAC TFs in DEGs have been upregulated after infection (Figure 8). Moreover, three unigenes (DN11118_c0_g2, DN844_c0_g1, DN844_c0_g2) annotated as NAC TFs were validated to get a high degree of homologous similarity with AtNAC066 by constructing phylogenetic tree (Supplementary Figure S9; Supplementary Table S13). Therefore, we hypothesized that the three NAC TFs might play a vital rol in resisting corm rot disease in saffron. Nineteen MYBs have been identified, of these twelve have been upregulated and seven have been downregulated. It has been reported that overexpression of *GhWRKY41* in *A. thaliana* and cotton can promote its resistance to *Verticillium dahliae* (Xiao et al., 2023). *WRKY38* and *WRKY62* in *A. thaliana* have been shown to be negative regulators of plant immunity and to be involved in the SA-mediated defense response (Kim et al., 2008). *WRKY8* has been reported to be involved in resisting tobacco mosaic virus (TMV-cg), by mediating ABA and ethylene signaling (Chen et al., 2013). Right now, we have obtained 17 *WRKY* TFs exhibiting significant expression difference compared to the healthy group (CsHG). Among of them, a WRKY member (DN11434_c0_g1) shares a high homology with GhWRKY41, thus implying its potential function of disease resistance (Supplementary Figure S9; Supplementary Table S13). In conclusion, all the selected TFs may form a vast regulatory network to coordinate plant growth and development as well as immune responses. However, the specific mechanism of the TFs participating in pathogen resistance in saffron remains obscure and needs to be studied further.

In summary, our study has screened the potential genes involved in corm rot disease and summarizes their gene expression pattern in saffron when it is susceptible to pathogen invasion, which provides effective data in exploring the molecular mechanism of disease resistance and breeding a new variety in saffron, with excellent corm rot resistance.

## 5 Conclusion

In present study, we revealed that the DEGs were predicted to participate in multiple signaling pathways to improve its corm rot resistance in saffron. Upon pathogen attack, both healthy and rot-infected saffron corms underwent rigorous transcriptional reprogramming of several biological processes, especially the activation of a large number of PTI, ETI, JA, SA, ABA-related genes, indicating that saffron corms employed multiple defense pathways to prevent the pathogen infection. Meanwhile, our results revealed that saffron corms initiated biotic and abiotic stress-responsive genes to resist pathogen stress. Candidate pathogen recognition genes obtained in this study are crucial for us to elucidate the molecular mechanism of corm rot resistance in saffron based on modern molecular biology and biochemical techniques, and it provides us an effective gene resource for breeding new variety of saffron owning disease resistance.

## Data Availability

The data presented in the study are deposited in the Genome Sequence Archive at the Beijing Institute of Genomics Data Center (https://bigd.big.ac.cn/), Chinese Academy of Sciences, accession number PRJNA1005600.
