# Supplementary material for: Mining of disease-resistance genes in Crocus sativus based on transcriptome sequencing
Source: Front Genet. 2024 Feb 2;15:1349626. doi: 10.3389/fgene.2024.1349626 (PMC10869511; doi:10.3389/fgene.2024.1349626)
Supplement: Supplementary file 1 [file Table2.DOCX]

Supplementary Material


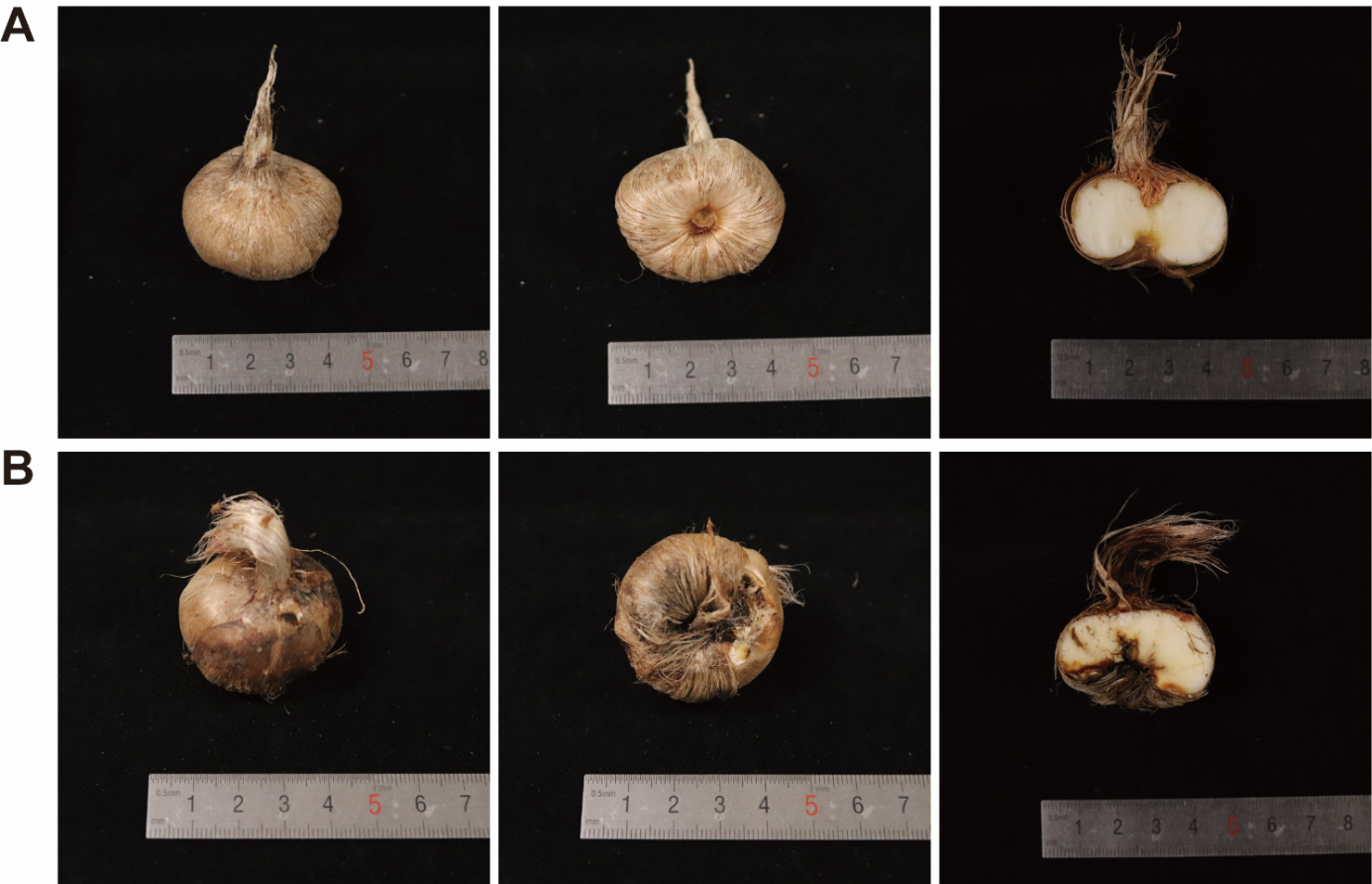


**Supplementary Figure 1**

Healthy and infected corms of *C. sativus*. **(A)** Healthy group (CsHG). **(B)** Infected group (CsIG).

**
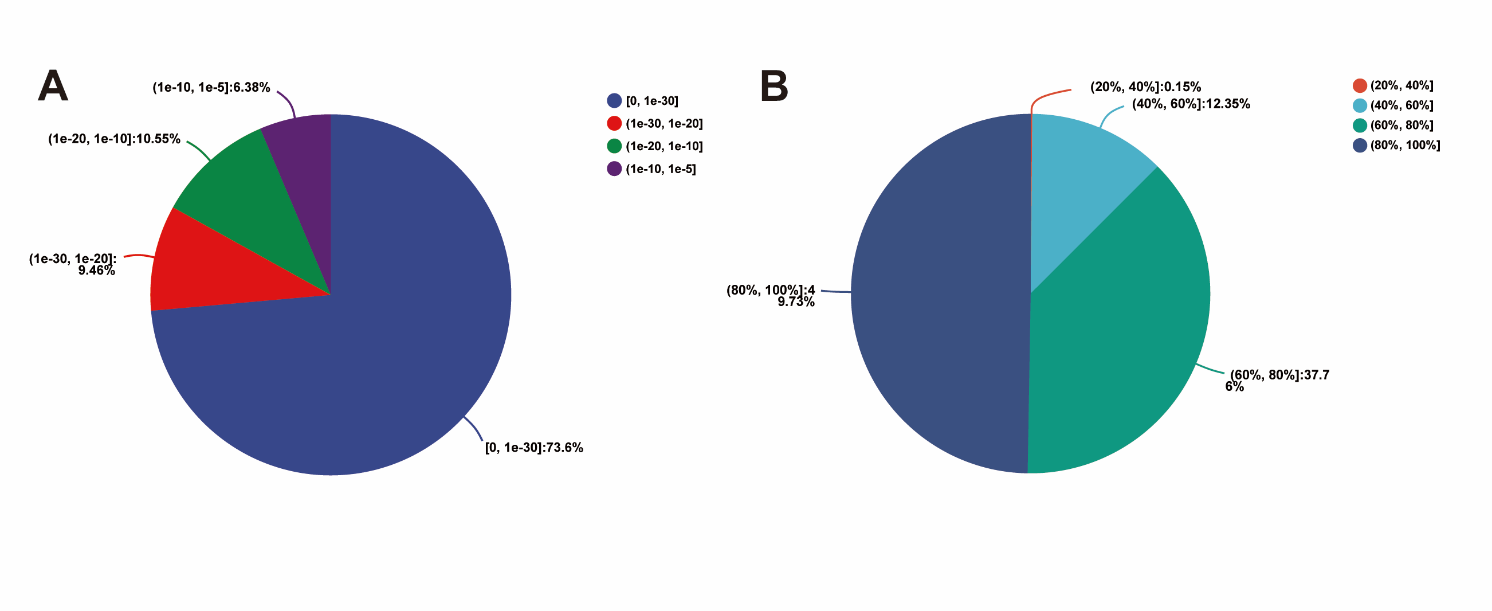
**

**Supplementary Figure 2**

Distribution of *E*-value and similarity based on NR annotation. **(A)** *E*-value. **(B)** Similarity.


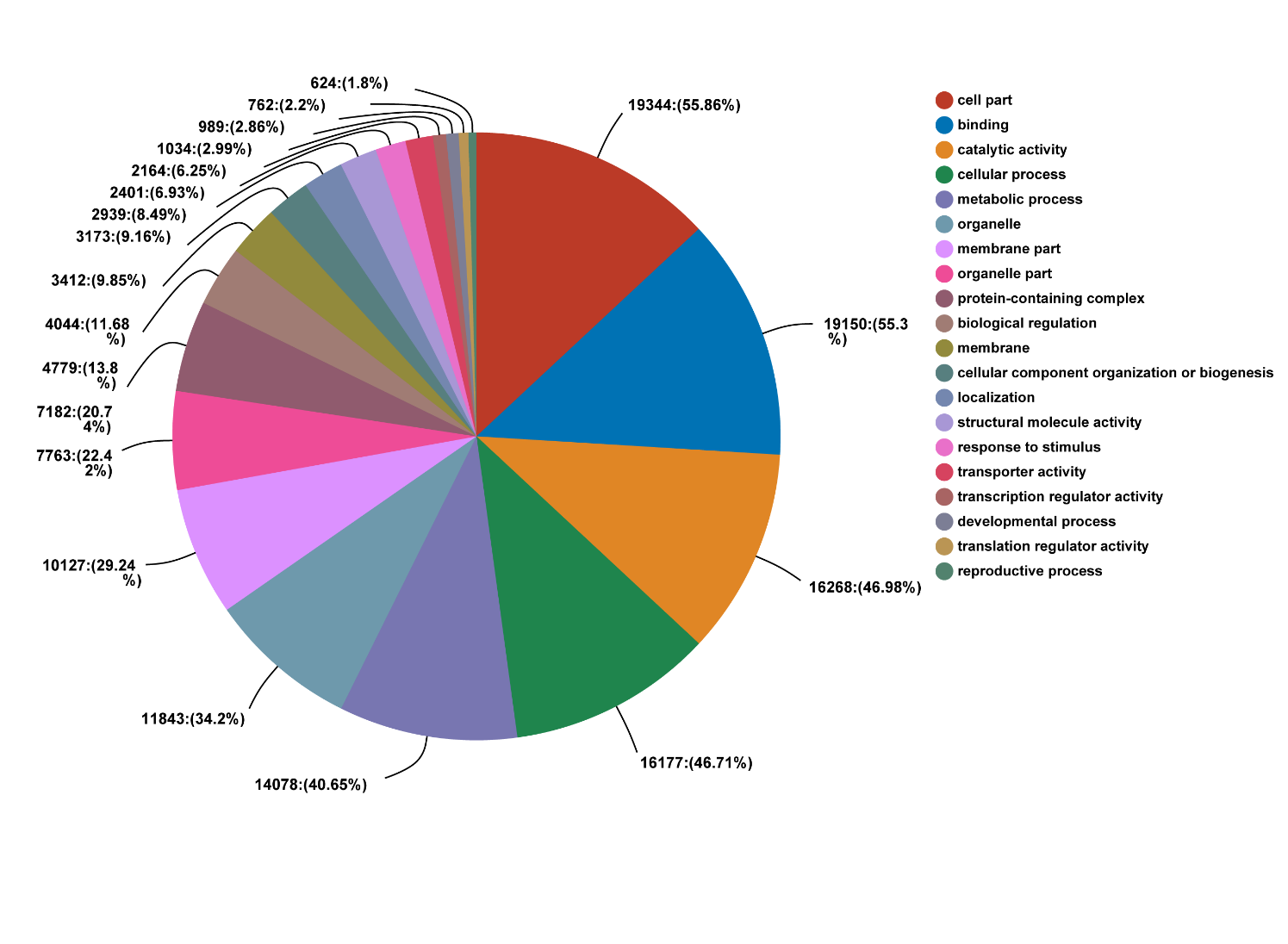


**Supplementary Figure 3**

GO functional classification of the unigenes.

**
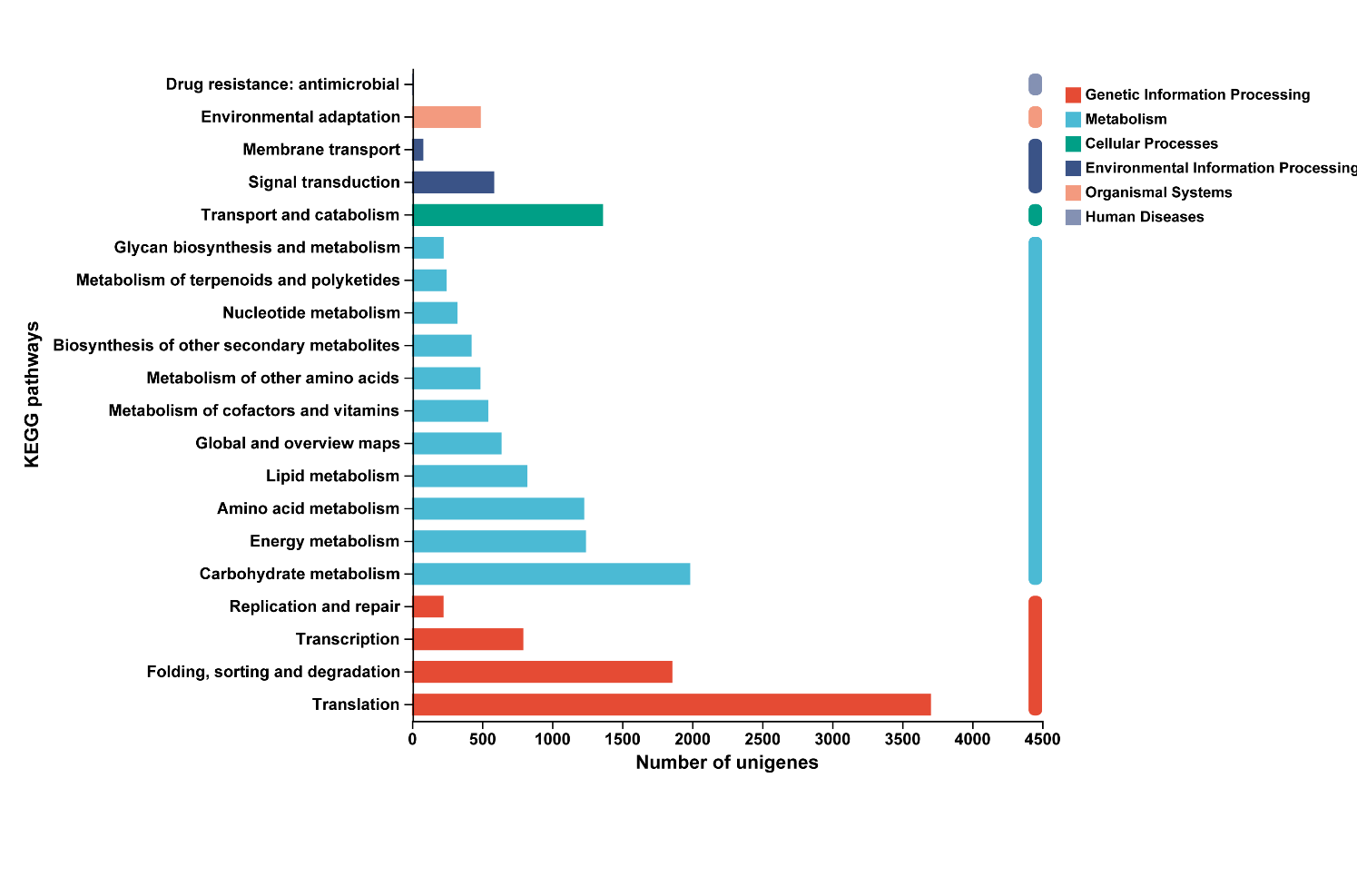
**

**Supplementary Figure 4**

KEGG-pathway classification of the unigenes.


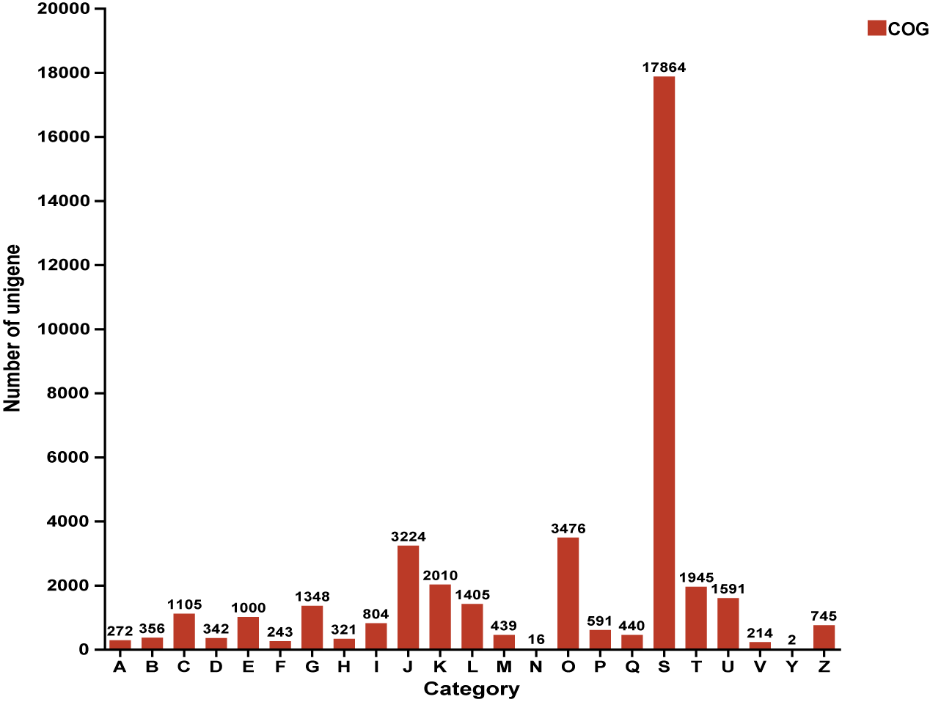


**Supplementary Figure 5**

COG annotations of the unigenes.


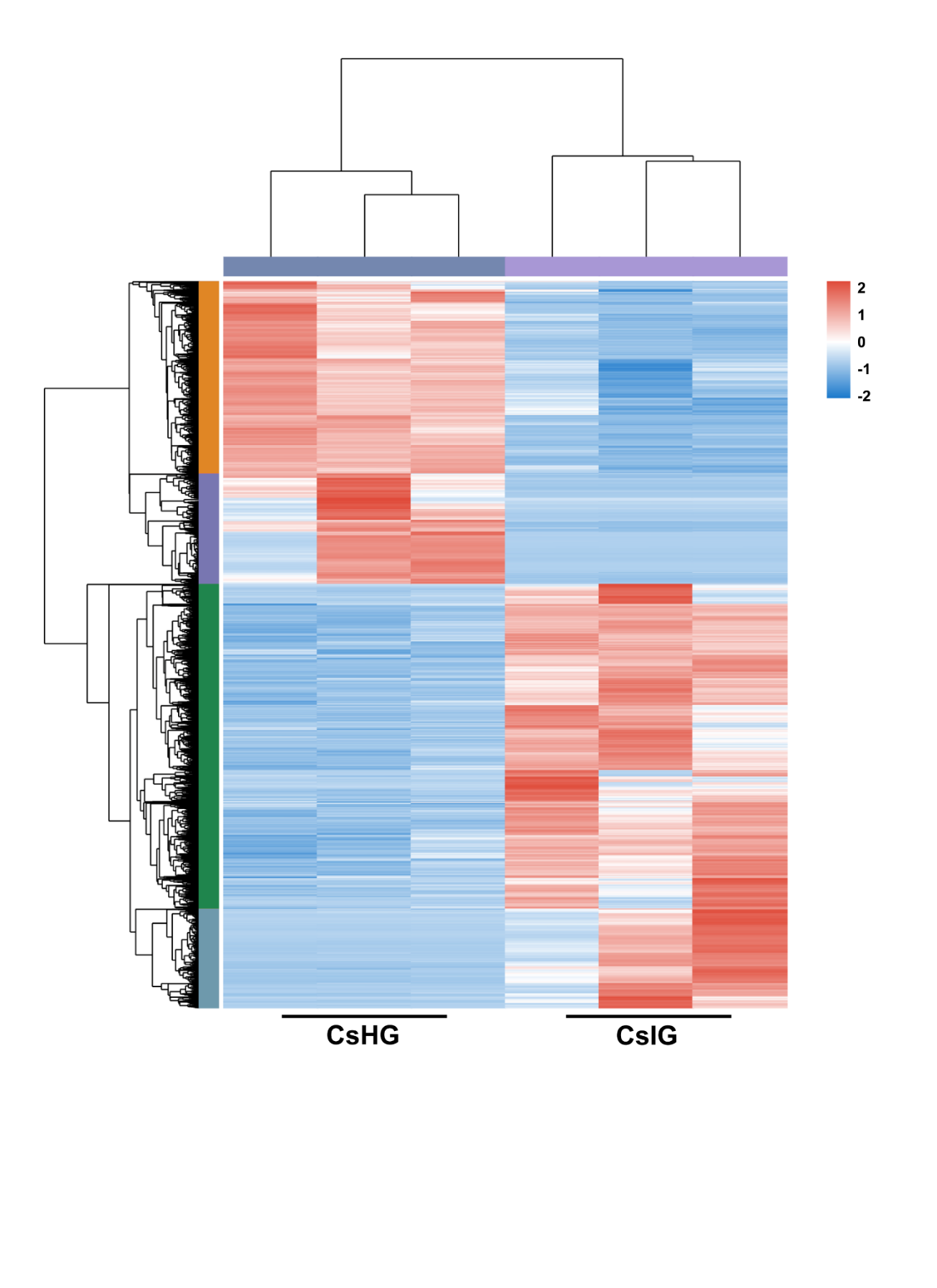


**Supplementary Figure 6**

Expression levels of differentially expressed genes in healthy and infected groups of *C. sativus*.

**
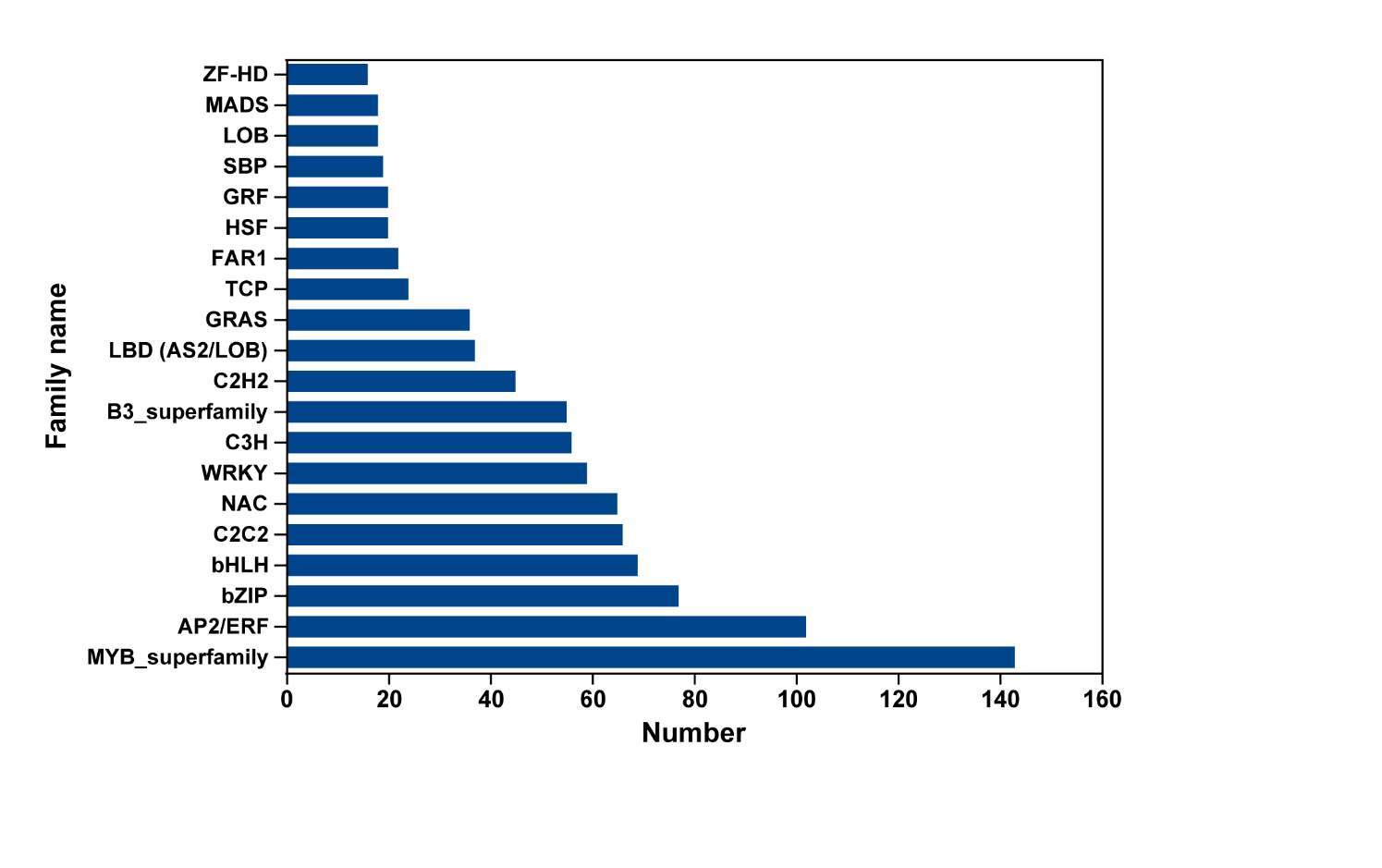
**

**Supplementary Figure 7**

Transcription factor families distributed in the transcriptome.


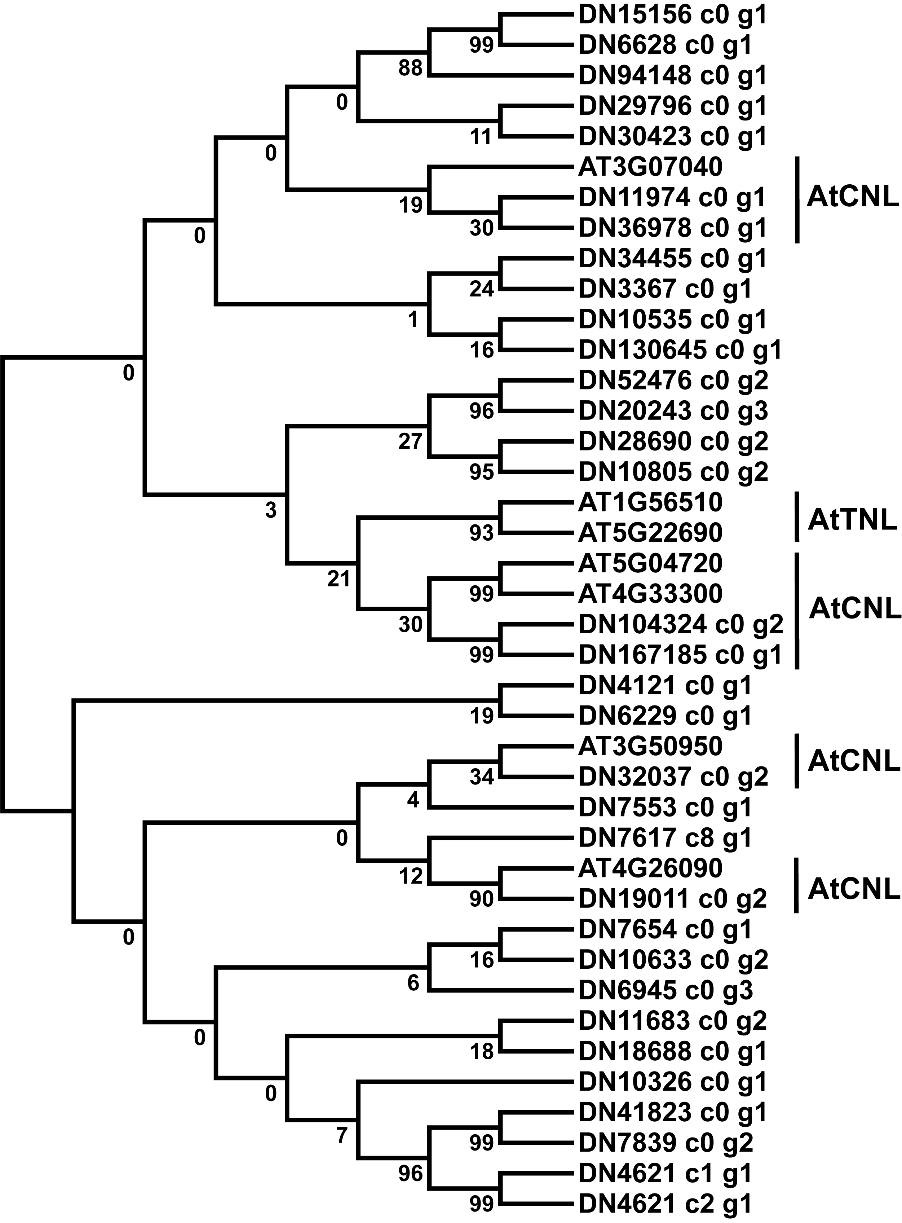


**Supplementary Figure 8**

Phylogenetic tree of differentially expressed PRGs containing the domain of CNL and TNL. AT5G04720, AT3G50950, AT4G33300, AT3G07040, AT4G26090 is the gene ID identified as CNL in *Arabidopsis thaliana*; AT1G56510, AT5G22690 is the gene ID identified as TNL in *A. thaliana.*


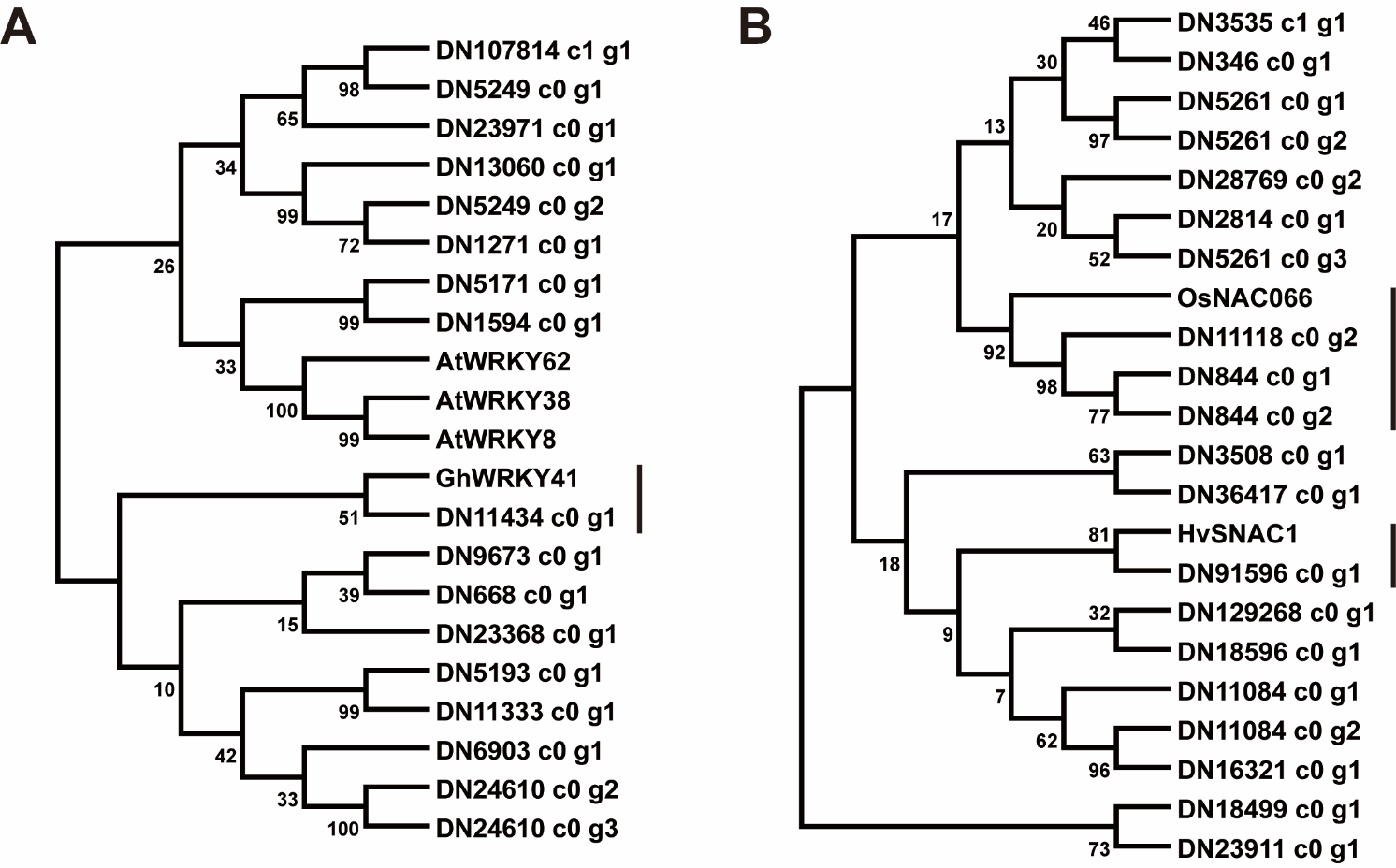


**Supplementary Figure 9**

Phylogenetic tree of differentially expressed WRKY and NAC transcription factors (TFs). **(A)** WRKY TFs. **(B)** NAC TFs.
